# Potential use of vibrational playbacks for management of pear psylla

**DOI:** 10.1093/jisesa/ieaf096

**Published:** 2025-11-07

**Authors:** Dowen Mae I Jocson, Louis B Nottingham, Tobin D Northfield, Elizabeth H Beers, Liesl Oeller, David W Crowder

**Affiliations:** Department of Entomology, Washington State University, Pullman, WA, USA; Tree Fruit Research and Extension Center, Washington State University, Wenatchee, WA, USA; Northwestern Washington Research & Extension Center, Washington State University, Mount Vernon, WA, USA; Tree Fruit Research and Extension Center, Washington State University, Wenatchee, WA, USA; Tree Fruit Research and Extension Center, Washington State University, Wenatchee, WA, USA; Department of Entomology, Washington State University, Pullman, WA, USA; Department of Entomology, Washington State University, Pullman, WA, USA

**Keywords:** acoustics, biotremology, vibrational communication, mating disruption, pest

## Abstract

Integrated pest management programs often use pesticides alongside behavioral tactics, such as mating disruption, to manage pests. Pest management using biotremology, the study of vibrations produced by organisms, is gaining attention but requires substantial knowledge of pests and their environment. Here, we built on previous characterizations of vibrational mating signals in pear psylla to assess if pear psylla (*Cacopsylla pyricola* Förster) communication behavior can be exploited for pest management. Specifically, we conducted greenhouse experiments to test the efficacy of 3 vibrational playback treatments for mating disruption: (i) control, (ii) white noise, and (iii) male mating signals, using 2 delivery methods: (i) plant substrate and (ii) trellis wire; these 2 methods assessed whether devices attached directly to pear saplings or trellis wire supporting saplings provided similar results. We also conducted experiments in pear orchards to assess effectiveness of vibrational playbacks as trap supplements. In the greenhouse, white noise and male mating signals delivered through plant substrates reduced pear psylla offspring in 1 of 3 experiments, but never when delivered through trellis wires. Sticky traps in orchards supplemented with vibrational signals trapped more adults and females than sticky traps alone. The results of this study suggest that pear psylla vibrational communication may be exploited for pest control and pest monitoring, but variable efficacy among experiments suggests a need for further examination into delivery methods.

## Graphical abstract

**Graphical Abstract:**
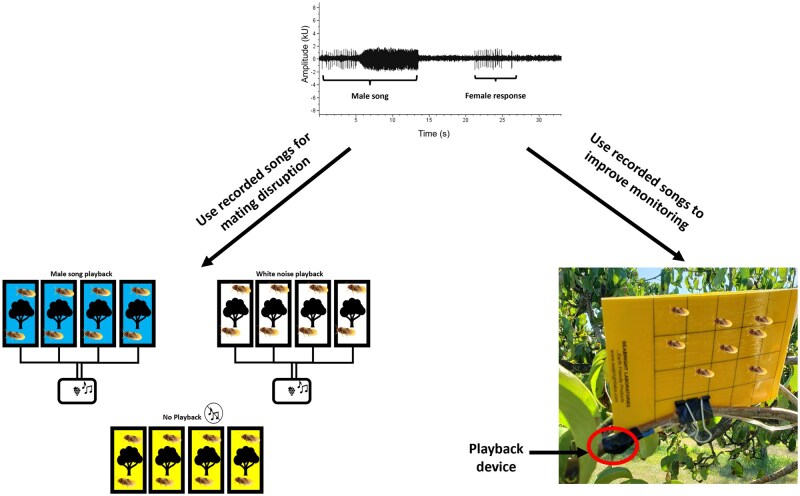

## Introduction

Integrated pest management (IPM) uses a diversity of tactics to manage pests (Whalon et al. 2008). Mating disruption is one such tactic, the process of preventing pests from finding mates. Many insects use chemical signals to find mates, and when signals are synthesized and deployed they confuse adults and delay mating through competitive mating disruption (Witzgall et al. 1999, Miller and Gut 2015). Efficacy of mating disruption using pheromones has been validated in numerous studies for pests such as codling moth (*Cydia pomonella*) (Lepidoptera: Tortricidae) (Thomson et al. 2001, Brunner et al. 2002, Witzgall et al. 2008). However, there are other forms of mating disruption that have received less attention. For example, noncompetitive mating disruption occurs when signals like sounds inundate the environment and overstimulate pest sensory mechanisms (Miller and Gut 2015). However, only recently have researchers begun to assess whether synthetic acoustic and vibrational signals can be used for mating disruption.

Many insects use vibrational signals for aggregation calls, alarms, and to attract mates (Cocroft and Rodríguez 2005, Randall 2010, Liao et al. 2022). Research in biotremology, the study of animal vibrations, includes the creation of mating disruption tactics using semiophysical signals (Hill and Wessel 2016, Hill et al. 2019). A prime example was conducted with American grapevine leafhopper, *Scaphoideus titanus* (Hemiptera: Cicadellidae), where playback of a male rivalry signal in grape vineyards reduced male signaling and male–female duets (Mazzoni et al. 2009). Vibrational signals may also be used for pest monitoring if they can be incorporated into traps. The first acoustic trap was used for mole crickets (Ulagaraj and Walker 1973, Walker 1982), and more recently one was created for brown marmorated stink bug, *Halyomorpha halys* (Hemiptera: Pentatomidae) (Polajnar et al. 2016b, Mazzoni et al. 2017a, Zapponi et al. 2023). Like pheromone traps, traps baited with acoustic signals may increase rates of pest detection.

Many factors go into the development of acoustic mating disruption tools, and detailed knowledge of pest behavior in response to vibrational playbacks is key (Mazzoni et al. 2017b, Krugner and Gordon 2018, Nieri and Mazzoni 2018). Acoustic signals have a variety of delivery mechanisms, such as speakers to transmit sounds or vibrational devices to mimic signal patterns produced by pests during mating; understanding how pests respond to unique signals is key to developing tactics that are effective in the field. For example, while vibrational signals might be attached directly to plant substrate, signals might be also transmitted along trellis wire for some crops (Polajnar et al. 2016a, Nieri et al. 2017, Campbell 2019). For a given pest and crop combination, testing various signals and delivery mechanisms to assess the most effective strategy is key to implementation within IPM programs.

Here, we assessed whether vibrational signals could disrupt a major pear pest, *Cacopsylla pyricola* (Förster) (Hemiptera: Psyllidae) (pear psylla). Pear psylla populations in Washington State United States are resistant to many pesticides (Harries and Burts 1965, Follett et al. 1985), causing growers to spend $3,750 per hectare on average for control (Nottingham et al. 2023). Pear psylla is most effectively controlled with insecticides combined with tactics such as tree washing, kaolin clay, and reflective ground covers (Brunner and Burts 1981, ­Nottingham et al. 2022), but there are no mating disruption strategies for this pest. Pear psylla males and females produce vibrational signals to attract and locate mates, and these signals have been synthesized in the laboratory (Jocson et al. 2023). Here, we tested whether these vibrational playbacks could affect pear psylla mating when transmitted both through the plant substrate and through a secondary substrate (trellis wire). We also explored whether vibrational signals could supplement sticky cards and provide more effective pear psylla traps. Overall, our study allowed us to test whether a novel mating disruption strategy had population level effects or could improve pest monitoring.

## Methods

### Insects Used in Experiments

In February 2020, 2021, and 2022, adult winterform psylla were collected by hand from pear orchards at the Washington State University (WSU) Tree Fruit Research and Extension Center (Wenatchee, WA, United States). In May each year, newly molted summerform adults were also collected. Pear psylla adults were placed on 1 m tall pear saplings (“Bartlett”) provided by Central Washington Nursery (Quincy, WA, United States) in 14 L pots with potting soil (Sunshine LC1, Sun Gro Horticulture, Agawam, MA, United States). Plants were kept in greenhouses (standard settings of 16:8 h, 21 to 24 °C:16 to 18 °C) at WSU (Pullman, WA, United States) in mesh rectangular cages (BugDorm-44590F, MegaView Science Co., Ltd, Taiwan), with ­winterforms and summerforms colonies kept separate. We used previously published adult call signals of summerform and winterform males that were captured and synthesized in a controlled laboratory (Jocson et al. 2023). Male signals consist of patterned chirps and trills, used to attract and find mates. While the interchirp intervals, number of chirps, and trill length do not differ significantly between winterform and summerform morphs, the summerform morph call frequency is around 1,250 Hz while the winterform calls between 600 and 900 Hz (Jocson et al. 2023). Using male calls as a signal is a form of “noncompetitive” mating disruption, where synthetic signals are used to flood the sensory environment of insects and confuse them (Miller and Gut 2015). This differs from using female signals, where males are attracted to “false females,” which is a form of competitive mating disruption (Miller and Gut 2015). Here we used male signals as they had been synthesized prior and are more complex than female signals, which only consist of chirps.

### Vibrational Playback in the Greenhouse

Our first greenhouse experiment tested if vibrational signals transmitted through pear stems affected mating of winterform and summerform psylla. We had 4 replicates of each of 3 treatments: (i) control (device attached but no signal), (ii) white noise (noncompetitive mating disruption), and (iii) male signal playback (competitive mating disruption). This experiment was conducted once with winterform psylla, starting on 16 March 2021, and twice with summerform psylla, starting on 19 May 2020 and 14 May 2021. Prior to each experimental run we removed male and female adults from the greenhouse colonies soon after molting and placed them in separate colonies to reduce chances of virgin adults mating prior to experiments.

For each replicate, we placed 5 female and 3 male adult pear psylla on a single pear sapling. We had a limited number of saplings, and these numbers were designed to be able to produce sufficient number of offspring for statistical analyses without overwhelming and killing the saplings, which were sensitive to stress in greenhouses (Jocson et al. 2023). Vibrational playback devices were made using a linear resonant actuator (Vybronics Inc.) soldered to radio wires and a stereo jack, plugged into audio interfaces (Tascam 4 × 4) connected to a Raspberry pi (Pi 4 Model B 4GB). This set up provided files of the vibration pattern for mechanical resonance of the plant material. These vibrational playback devices were attached to the main pear stem, 2 cm above soil. For the male signal playback treatments, we used recordings of a summerform male at 25 °C or a winterform male at 21 °C, depending on the experiment. White noise was produced with the Audacity program and contained the range of frequency in the human audible sound spectrum (20 to 20,000 Hz). This range covers the fluctuation in frequencies produced and received by pear psylla (Jocson et al. 2023). We standardized the playbacks by setting the output volume to the playback devices at the same level (50% on the audio interface), which was the only method available to filter the signal noise. Prior experiments showed that these volumes were sufficient to produce behavioral responses in psylla within a realistic signal environment (Jocson et al. 2023). For example, while these signals have been shown to alter mating success, we have observed that males and females engage in typical behaviors, with males searching and females largely stationary and receptive, regardless of the signal treatments. After 28 d of continuous experimental playback at a constant frequency, we collected 5 random cuttings (5 to 7 leaves with 15 to 20 cm of attached stem) from each plant and froze them to terminate development of pear psylla eggs and nymphs; these individuals were counted as a measure of reproductive success. We also aspirated adults from the cuttings and counted them.

### Trellis Vibrational Playback

Our second greenhouse experiment tested whether signals transmitted through trellis wire affected pear psylla mating. In this experiment, we tested if direct transmission of the signal to the plant, or delivery to multiple plants connected to a central trellis wire, affected mating. There were 3 playback treatments: (i) control (device attached but no signal), (ii) white noise (noncompetitive mating disruption), and (iii) male signal playback (competitive mating disruption), along with 5 distance treatments (0, 0.6, 1.2, 1.8, and 2.4 m from playback device); there were 2 replicates of each treatment × distance combination with summerform pear psylla.

For each replicate, on 13 July 2021, 5 pear saplings were arranged linearly along trellis wires at 0, 0.6, 1.2, 1.8, and 2.4 m from a vibrational playback device attached to the trellis wire. Five female and 3 male adults were placed on each plant. As with the prior experiment, signals were played continuously for 28 d, after which 5 random cuttings (5 to 7 leaves with 15 to 20 cm of attached stem) from each plant were frozen to terminate development of pear psylla eggs and nymphs. We also aspirated pear psylla adults from the cuttings. We counted the number of individuals from each life stage (egg, nymph, adult) from the cuttings and aspirated adults.

### Vibrational Playback Supplemented Sticky Traps

There were 5 replicates of 2 treatments: (i) control (playback device but no signal) and (ii) female signal playback (competitive mating disruption); the experiment started 12 April 2021 when the first summerform adults were emerging at the WSU Tree Fruit Research and Extension Orchard (Wenatchee, WA, United States). We randomly placed yellow sticky cards on 10 pear trees, with one sticky card per tree on a branch 180 cm from the ground. Five traps were supplemented with the same vibrational playback devices as prior experiments but played female response signals through the stem, while control traps had no playback devices. Playback devices were left playing continuously for 48 h, after which we counted and determined the sex for all pear psylla adults caught.

### Statistical Analysis

We used generalized linear models with a negative binomial distribution to account for overdispersion (MASS package, R v. 4.2.1) to assess (i) effects of signal treatments on offspring number in the vibrational playback experiment and (ii) effects of signal treatment, distance from signal, and their interaction on offspring number in the trellis experiment. For each analysis, separate models were created for summerform and winterform psylla. We hypothesized that male signals and white noise would both impair mate finding, resulting in fewer offspring than psylla not exposed to signals. Our design also allowed us to test the strength of competitive (male signal playback) and noncompetitive (white noise) mating disruption on psylla mating.

Efficacy of supplemented sticky cards was measured by the number of adults caught. We used a generalized linear model with a Poisson distribution (MASS package, R v. 4.2.1) to test if sticky traps with a female playback caught more adults than a sticky trap alone. We also used a generalized linear model with a logistic distribution to assess the sex ratio of the adults caught. For all models we used Tukey Honestly Siginificant Difference (HSD) tests to separate means for significant treatments.

## Results

### Greenhouse Vibrational Playback Experiment

**Fig. 1.**
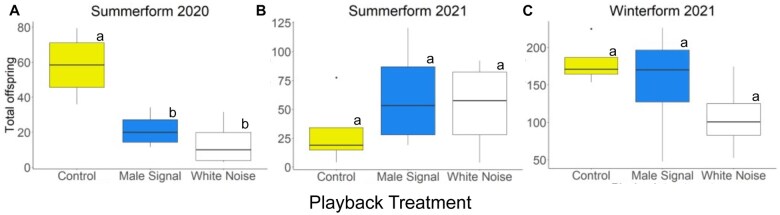
Summerform experiments from (A) 2020 and (B) 2021 and (C) winterform experiments from 2021 showing effects of treatments on total pear psylla offspring. In the 2020 summerform experiment (A), white noise and male signal treatments had significantly less offspring than controls; in the other 2 experiments (B and C), there were no significant differences among treatments. Statistical differences between treatments were determined using Tukey’s test and are noted by lowercase letters (a, b).

In the summerform 2020 experiment the treatments significantly affected the number of pear psylla (χ^2^ = 15.7, df = 2, *P *< 0.001), with white noise (*Z *= −2.61, *P *= 0.023) and male signal (*Z *= −3.73, *P *< 0.001) treatments having fewer pear psylla than the control (Fig. 1A). In contrast, the summerform 2021 experiment had no significant effects of treatments (χ^2^ = 1.49, df = 2, *P *= 0.48) (Fig. 1B). Similarly, we found no effects of treatments on the number of pear psylla offspring for the winterform 2021 experiment (χ^2^ = 3.50, df = 2, *P *= 0.17) (Fig. 1C).

### Greenhouse Trellis Vibrational Playback Experiment

**Fig. 2.**
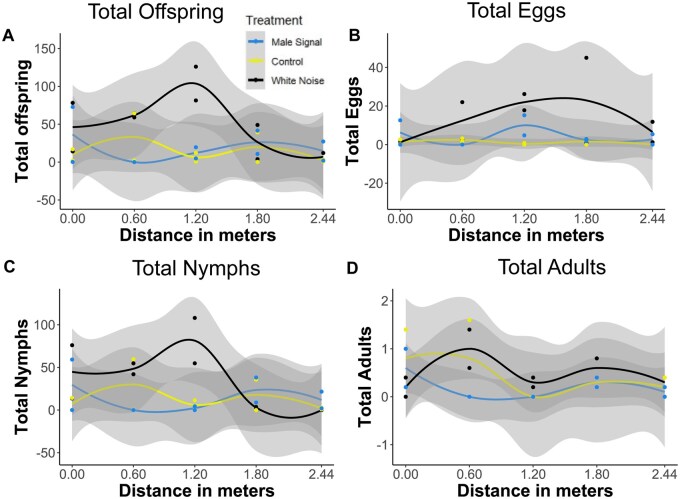
Results from the trellis experiment showing the total number of offspring (A), eggs (B), nymphs (C), and adults (D) on pear plants by playback treatment across the various distances from the source tested. Gray bands represent a loess model fit with a 95% confidence interval.

Vibrational playbacks along a trellis had a marginally significant effect on the total number of psylla offspring (χ^2^ = 5.38, *P *= 0.07), with the white noise treatment having the most total offspring (Fig. 2A). The effects of treatment were highly significant on the number of pear psylla eggs (χ^2^ = 15.3, *P *< 0.001; Fig. 2B) but not on the number of pear psylla nymphs (χ^2^ = 2.61, *P *= 0.27) or adults (χ^2^ = 1.30, *P *= 0.52) (Figs 2C and D). The experiments showed that these effects were consistent across distances (all distance and treatment × distance effects had a *P *> 0.20).

### Vibrational Playback Supplemented Sticky Cards in Orchard Pear Trees

**Fig. 3.**
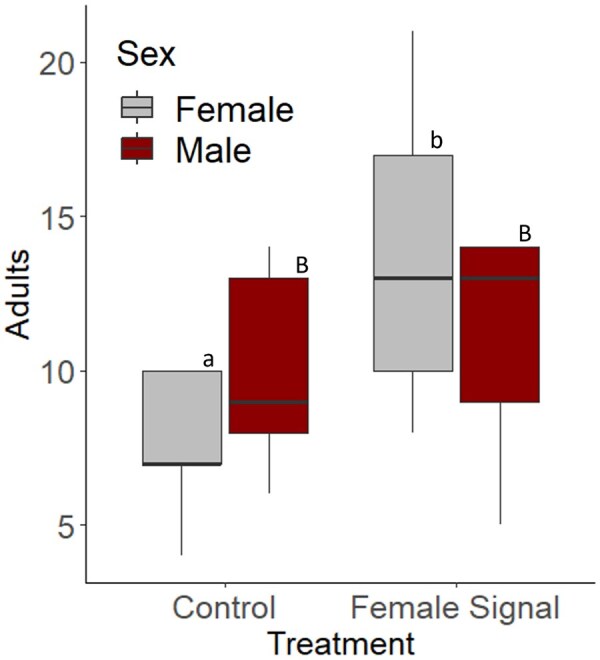
Adults caught on yellow sticky cards supplemented with vibrational female signals (or not supplemented). Data were separated by the sex of the adults caught. Marginally more adults, specifically more females were caught on the cards supplemented by the female vibrational signal (*P *= 0.07). Statistical differences were determined using Tukey’s test and are noted between females by lowercase letters (a, b) and males by uppercase letters (A, B).

Sticky cards supplemented with female response vibrational playbacks had significantly more adults caught compared to sticky cards without signals (*Z *= −2.10, *P *= 0.036; Fig. 3). Post hoc analyses showed that there were also marginally more females caught on sticky traps with vibrational playbacks than without vibrational playback (*Z *= 2.43, *P *= 0.07; Fig. 3).

## Discussion

Pear psylla and many closely related insects use vibrational signals to attract and locate mates (Cocroft and Rodríguez 2005, Liao et al. 2022). Winterform female psylla overwinter on alternative hosts adjacent to pear orchards and return to pear in the spring to search for mates (Krysan and Horton 1991, Cooper et al. 2019). Due to this behavior, spring is potentially an ideal time to deploy vibratory signals to disrupt mating behavior. Disruption of mating behavior may delay oviposition to suboptimal times or locations and thus decrease overall reproductive success (Fitzpatrick 2006, Kawazu et al. 2014, Wang et al. 2021). Our experiments show that vibrational playbacks, depending on type of signal and delivery method, may reduce the number of pear psylla offspring. However, it is more likely that acoustic signals might benefit monitoring and trapping programs by improving trap captures as opposed to being an areawide control tactic. For all our experiments, we were only able to conduct a relatively small number of replications due to logistical and other constraints. Our results should thus be viewed as largely preliminary, where more replication in the future would provide more robust conclusions.

Vibrational playback of male signals and white noise through plant substrate reduced pear psylla reproduction in the plant shoot-based experiments, but not in experiments with trellis wire. Moreover, only summerform psylla in 2020 showed a strong response, while our experiments in 2021 were not significant. While it is unclear what caused this variation, reproductive output was considerably higher in 2021 than 2020, and populations were 2 to 3 times as large. It is possible that mating disruption was most effective at low levels of reproductive output. In addition, male signals also only reduced pear psylla offspring in the summerform generation, which might limit the effectiveness of the strategy given that early-season winterform psylla are the primary target of mating disruption. Nonetheless, our results build on literature suggesting playbacks of mating signals through plant substrates can alter pest behavior in ways that reduce reproduction, but only when consistently applied (Hofstetter et al. 2014, Hill and ­Wessel 2016).

Our trellis experiment was similar to studies with leafhoppers in grapes, where male signals were delivered through trellis wires and reduced male signaling and male–female duets (Polajnar et al. 2016a). However, male signal playbacks through trellis wire were not effective at reducing offspring production by pear psylla. Unlike grapevines, pears have more woody material to reverberate, and vibrations sent through trellis wires may lose energy quickly. Substrate property and shape affect transmission and propagation of signal vibrations, which can create reflected waves that distort the signal (Michelsen et al. 1982, Keuper and Kühne 1983, Čokl and Virant-Doberlet 2003). This could explain why we might have lost the effectiveness of white noise at the middle distance. We also did not filter out background noise from male signal recordings, which may have resulted in signals perceived as inauthentic (Michael et al. 2019). While currently most pear orchards in the United States are not trellised, exploring this as a method to deliver vibrations at scale is a step in determining the future of mating disruption for pear psylla.

Audio playbacks of mating signals have also been shown to improve traps for pest surveys (Mankin et al. 2013, Zapponi et al. 2023). Trap efficacy is dependent on density and activity levels of insects and placement and attractiveness of the trap itself (Horton 1994, 1999). Because insects are less likely to be captured when at low population densities or when too few traps are deployed, increasing the attractiveness of traps could improve monitoring abilities under these less favorable scenarios (Horton 1994, 1999). Moreover, using sticky traps for pear psylla tends to result in captures biased towards males due to male searching behavior, and may thus underestimate total population size (Horton 1993, Horton and Lewis 1997). Our results showed playback devices addressed both issues, as traps paired with acoustic signals caught more psylla and more females than traps without signals. Given the relatively weak effects of acoustic signals on the number of psylla offspring, our study suggests that the primary immediate utility of acoustic signals may be to improve traps for psyllid monitoring rather than for population suppression.

We hypothesized that males would be more attracted to acoustic traps because they were baited with female acoustic signals, but our results showed the opposite. It is unclear why this occurred, but a study that used brown marmorated stink bug female signals to enhance trapping also unexpectedly showed increased numbers of females (Zapponi et al. 2023). Female mating rivalry behavior is often overlooked, and competition among females may lead to signals attracting individuals of the same sex (Čokl et al. 2017, Zapponi et al. 2023). While our study did not investigate whether traps reduced female density in the broader field (and it is unlikely they did, given that we placed few traps in the field relative to the total number of trees in a production orchard), traps with acoustic baits may improve pear psylla monitoring.

Pear variety may also play a role in reproductive success of pear psylla. There are at least 24 species of *Cacopsylla* that use pear as hosts (Cho et al. 2017) so *C. pyricola* may have a host preference among varieties found in the Pacific Northwest United States. Anjou has been noted for its high vigor (ie produces more shoots) (Pasa and Einhorn 2014), and pear psylla prefer to oviposit on young foliage (Pfeiffer and Burts 1983, Horton 1990), which may explain high numbers of offspring in Anjou arenas in both the winterform and summerform experiments. Possibly because of these higher psylla populations, scientists have observed that Anjou pears are more susceptible to pear psylla damage than Bartlett. This makes Anjou a good host for pear psylla where mating disruption may be most effective at reducing populations.

Our studies show vibrational playbacks have potential for improving traps and may also aid in disrupting pear psylla mating. However, application of vibrational playbacks added to sticky traps or for mating disruption needs more widespread testing, particularly to uncover factors that can reduce efficacy (for both experimental and implementation scenarios). Future studies may need to employ a larger testing area, such as caging trees or blocks with or without signals. Despite the caveats, our results show promise in exploiting pear psylla communication systems for both improved monitoring and control. Our results also build on the literature from the field of biotremology to suggest vibrational playbacks through plant substrates can be an effective method of altering pest behavior in ways that reduces reproduction (Mazzoni et al. 2009, Hofstetter et al. 2014, Hill and Wessel 2016). We anticipate further studies and IPM programs will begin to implement acoustic signals to enhance the effectiveness of monitoring traps and disrupt mating behavior to suppress insect populations in cropping systems.
